# Plague and the I.M.S.

**Published:** 1919-08-16

**Authors:** 


### Plague and the I.M.S.

The recent outbreaks of plague in India, as well as the isolated cases occurring in this country, bring home very forcibly the fact that we have still much—indeed, almost everything that is vitally important—to learn concerning the etiology of this disease. We know of the spread by rats, etc., but it becomes increasingly apparent that many other factors, such as atmospheric, geographic, and racial conditions, may have a marked influence on the severity and spread. There is an enormous field of research here opened up, and in our Indian Medical Service we have the organisation located and equipped for its performance, but, curiously enough, we see attention first drawn to the need of Government assistance and support in the pages of an American journal! Certainly there has been some recent improvement in the conditions of pay, but, as is pointed out, work is heavy and holidays hard to obtain. The I.M.S. is ideally placed for such investigations, penetrates to the remotest sources of the disease, and contains many of the finest minds which could be set to tackle the problem of a disease which levies so heavy a toll on the manhood and prosperity of the Empire, but it is not to be expected that the future can hold promise for a service treated in ungenerous terms. Their pay is worth little more than half what it was at the beginning of the century, and, as our contemporary points out, if this lack of funds and encouragement be not remedied, apart from the unlikelihood of valuable research being done, the great medical schools will hardly continue to advise their students to seek the Indian Army as a career valuable to themselves and the Empire.

